# The Effect of Novel Low-Dose Caffeine Products on Physical Performance

**DOI:** 10.3390/nu18050791

**Published:** 2026-02-27

**Authors:** Andrew Thomas Hulton, Isobel Staines, Oscar Clark, Arun Subramaniam, James Matt Green

**Affiliations:** 1 Discipline of Nutrition, Exercise, Chronobiology and Sleep, University of Surrey, Guildford GU2 7XH, UK; 2 Department of Kinesiology, University of North Alabama, Florence, AL 35632, USA

**Keywords:** caffeine, ergogenic, low dose, strength endurance, perception of effort

## Abstract

**Background**: Caffeine is an ergogenic aid shown to delay fatigue, increase arousal, and improve performance. Recommended doses are 3–6 mg/kg BM, although evidence supports lower doses (<3 mg/kg). Some conflicting results have highlighted that lower doses may still be ergogenic, and with new pouch and gum products, further research is warranted. **Method**: This study investigated the effects of novel low-dose caffeine products on muscular endurance, strength, and power. A repeated-measure, crossover design (pouch 80 mg, gum 80 mg, control gum 0 mg) was employed, recruiting nineteen participants (age 22.4 + 4.8 yrs; weight 72.8 + 16.9 kg; relative caffeine dose 1.1 mg/kg). Participants completed a battery of tests, including 60% 1 RM single leg press (LP) and shoulder press (SP) to exhaustion, counter-movement jump, and isometric mid-thigh pull, in addition to providing ratings of perceived exertion (RPE) during endurance tests. One-way repeated measures ANOVA was conducted on all measures associated with physical tasks, with a two-way repeated measures ANOVA conducted for RPE. **Results**: No significance was observed among conditions for physical tests. However, effect sizes, employing Cohen’s D classification, identified a moderate (d = 0.55) and small (d = 0.45) effect for the caffeine pouch compared to the placebo and caffeine gum for the LP respectively. Further, small effects for the pouch compared to the placebo were observed (d = 0.33) for the SP. Significant differences were produced for RPE during the LP (*p* = 0.022), with post hoc analysis identifying significant differences between the placebo vs. caffeine pouch (*p* = 0.032). **Conclusion**: Low-dose caffeine has the potential to produce meaningful effects on strength endurance, likely linked to caffeine mechanisms reducing RPE.

## 1. Introduction

Caffeine, C8H10N4O2, belongs to the methylxanthine class and is a widely consumed central nervous system (CNS) stimulant [1], with over 90% of adults reporting regular intake [2]. It is a well-established ergogenic aid employed to enhance sporting endeavors in training and competition [3], with initial investigations [4] suggesting the ergogenic effect was attributed to an increased fat oxidation and sparing of the limited glycogen stores. However, findings have identified ergogenic effects when muscle glycogen was not a limiting factor [3], and therefore the primary mechanism supported is caffeine’s adenosine antagonism [5], blocking the inhibitory effects of adenosine and subsequently increasing the neural firing and heightened release of excitatory neurotransmitters [6]. These neurological effects translate into delayed fatigue, heightened alertness, and faster reaction times. Furthermore, caffeine has been found to increase calcium release and uptake from the sarcoplasmic reticulum, improving excitation–contraction coupling and muscle power output [7]. Caffeine’s ability to suppress muscular fatigue during exercise may also contribute to improved endurance performance by allowing athletes to maintain higher intensities for longer periods [8,9,10]. These mechanisms collectively support the potential ergogenic effects of caffeine on endurance, strength, and power exercises.

Caffeine’s use within athletic populations is widespread [11,12,13], and optimal dosing strategies range between 3–6 mg/kg BM [3], equivalent to approximately 200–400 mg for the average adult. However, these moderate-to-high caffeine doses may heighten the prevalence of side effects, with a recent review article by de Souza and colleagues [14] suggesting that the incidence and magnitude of side effects, such as gastrointestinal distress, nervousness, mental confusion, and inability to focus [15], was greater in high caffeine dosage than lower. Therefore, investigations into low-dose caffeine consumption have increased, and though conflict still exists, with the literature demonstrating that moderate doses (3 mg/kg BM) were required to enhance power output over low caffeine doses (1 mg/kg BM) [16], there is growing evidence that low caffeine doses (<3 mg/kg or ~100–200 mg) may impart ergogenic benefits [17]. Furthermore, a meta-analysis [18] stated that the effects of caffeine consumed in very small doses (0.9–2 mg/kg) can still induce an ergogenic effect on muscular strength, muscular endurance, and mean velocity, with the magnitude similar to that of higher doses. While overall results are still equivocal, findings indicate a potential for doses under 200 mg to produce meaningful improvements on performance, especially in recreationally active individuals. In addition to the dose, it is widely accepted that responses can be very personalized to how individuals absorb, metabolize and utilize caffeine within the body [3]. Yang and colleagues [19] highlighted genes CYP1A2 and potentially ADORA2A as partly responsible for the inter-individual differences observed with caffeine metabolism.

As well as the variations in the caffeine dose and individual responses, there are also several ingestion method alternatives to traditional drinks, including chewing gum, mouth rinsing, nasal sprays, gels, bars, and capsules/powder. Caffeine gels seem to be popular amongst athletes participating in endurance sport, with approximately 60% of athletes in an observational study reporting caffeine use in this format [20]. Of further interest, the gels were typically consumed containing a lower dose than the previous dose implicated as necessary for ergogenic benefit, highlighting the use of low doses and the need for further research in this area. Additionally, a growing ingestion method is caffeinated chewing gum. This format has gained traction due to research stating that caffeine is absorbed quicker through the buccal mucosa compared to gut absorption [21], accelerating its ergogenic properties. Within the first 5 min of mastication, 85% of the caffeine is released, with peak plasma concentration occurring after only 15 min, whereas methods that rely on gut absorption see peak concentration after approximately 45–60 min [21]. This increased speed of absorption provides athletes with more scope to consume caffeine closer to exercise conception, during the exercise, and prior to sporting involvement when timings may not be pre-planned. Early investigations have shown promise for improving endurance performance with caffeinated gum [22,23], supported by a systematic review who reported caffeinated chewing gum to have positive effects on improving athletic performance [24]. Furthermore, the review by Yang and colleagues [24] examined the effects on strength and found performance enhancements for lower limb strength with lower doses (2 mg/kg) than traditionally recommended. In addition, in agreement with previous reports [18], an investigation with highly trained soccer players produced significant results for quadricep strength with a lower dose of 100 mg [25]. Subsequently, a new low-dose caffeine (80 mg) product, which has yet to be investigated, is the oral caffeine pouch. This method contains the caffeine in a pouch that is made of plant-derived cellulose, similar to the material used commonly in tea bags. The pouches are placed between the upper lip and gum, which is ideal for the caffeine to absorb via the buccal mucosa akin to gum. The caffeine pouch, like caffeine gum, may be a valuable alternative due to the potential to reduce the gastrointestinal side effects compared to liquid forms by bypassing the gastrointestinal tract yet still providing a caffeine dose that may be ergogenic.

Therefore, this investigation compared the ergogenic effects of low doses of caffeine on measures of muscular endurance, strength and power. The caffeine was delivered in novel formats of oral pouches and chewing gum, compared to a non-caffeine placebo chewing gum. By evaluating these physical parameters this investigation can broadly assess the ergogenic value of a 80 mg caffeine dose for physical work. We hypothesize that both low-dose caffeine products will enhance muscular endurance, strength and power, though interindividual variability may be observed. Low-dose caffeine products have the potential to enhance physical performance, which may provide a safe and practical alternative strategy for recreational athletes and those seeking to improve overall performance.

## 2. Materials and Methods

### 2.1. Participants

Nineteen healthy, recreationally active adults (11 males, 8 females; mean + SD age 22.4 + 4.8 yrs, weight 72.8 + 16.9 kg) were recruited for this investigation. Participants met the inclusion criteria of the age range of 18–45 years old, non-smokers, physically active with an exercise frequency of ≥2 days/week, and not pregnant at the time of participation or with any current musculoskeletal injuries, medical conditions or medications that could interact with caffeine. Potential participants were excluded if they were currently injured or undergoing any treatment for medical conditions that were likely to affect their physical performance. Participants were fully informed of all testing procedures, purposes, and risks verbally and in writing. Participants completed a Physical Activity Readiness Questionnaire [26] and provided written consent prior to participation in the investigation, which had received favorable ethical opinion from the University of Surrey Ethics Committee (ref: FHMS 22–23 172 EGA) and was conducted in accordance with the Ethical Standards in Sport and Exercise Science Research: 2020 Update [27].

### 2.2. Study Design

This study employed a block randomized, double-blind (for gum conditions only), crossover design to investigate the effects of low-dose caffeine (80 mg) in the form of chewing gum and oral pouches on physical performance in healthy, recreationally active adults. Participants were required to complete four visits in total, one familiarization and three experimental trials, with visits at least 48 h apart. Each visit lasted approximately 45 min, with participants required to refrain from exercise and alcohol consumption 24 h before each visit and caffeine consumption for 12 h. The investigation used two novel methods of caffeine delivery: caffeinated chewing gum and oral pouches. These methods were chosen as they allow for rapid absorption of caffeine through the buccal mucosa, which is highly vascularized [21]. The pouch is placed between the lip and gum, where it mixes with saliva, allowing the caffeine to be absorbed, although it must be stated that absorption rate has not been measured for these pouches.

### 2.3. Supplementation Protocol

Prior to the first exercise, participants either placed (1) an 80 mg caffeine pouch (Citrus Tang flavor, BELTER, London, England) between their gum and top lip for 15 min, (2) chewed an 80 mg caffeinated chewing gum (Peppermint flavor, Vyper Caffeine Gum, Caivano, Italy) for 15 min or (3) chewed a 0 mg placebo gum (Peppermint flavor, Wrigley’s Extra, England) for 15 min. Both gums were mint-flavored and had a similar shape to support the blinding process. However, at the time of this investigation, there was no placebo pouch available that could be included. Participants were aware that the pouch contained caffeine and that only one of the chewing gum conditions contained caffeine to try and include some blinding within the investigation. The mean relative caffeine intake equated to 1.1 mg/kg BM. All gums and pouches were removed prior to exercise.

### 2.4. Procedure

Familiarization Trial: The primary aim of the initial visit was to ensure that participants understood the structure of the experiment. This involved a 5 min dynamic warm-up, determining a 1 repetition maximum (RM) to calculate 60% of 1 RM to focus on muscular endurance, and familiarization with the physical tests. The 1 RM testing followed a standardized protocol, with participants performing warm-up sets with progressively heavier loads followed by maximal attempts with increasing weight until they were unable to complete a single repetition with correct form. Adequate recovery (3–5 min) was provided between maximal attempts to ensure optimal and accurate performance [28]. The physical tests included a single leg press and shoulder press to exhaustion at 60% of the individualized 1 RM, isometric mid-thigh pull (IMTP), and a counter-movement jump (CMJ).

Throughout the familiarization trial, researchers monitored participants closely to ensure they were following the instructions correctly and using the correct technique. Participants practiced each test in the order of the experimental trials until they felt confident and comfortable with the procedures.

**Figure 1:**
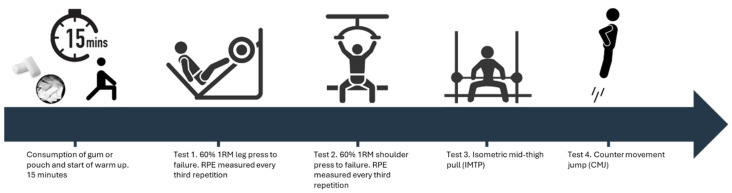
Schematic of the testing proceedings.

Experimental Trials: The remaining three visits were the experimental trials, which involved administering one of the conditions (1. caffeine pouch, 2. caffeine gum, 3. placebo gum) in a counterbalanced order. No caffeine was consumed on the day prior to the experimental trials, and all participants completed their trials at the same time of day to avoid any circadian variation that may affect performance [29]. Depending on the assigned condition, participants chewed the assigned gum or inserted the pouch under their top lip for 15 min. This aligned to the rapid absorption kinetics of chewing gum, with the onset of action occurring as quickly as 5–10 min following administration, even though peak caffeine concentration occurs much later [21], and was in support of previous investigations who observed performance improvements when caffeine was administered close to the onset of exercise compared to 60 or 120 min prior to the event [22]. A standardized warm-up was completed that included light aerobic cycling followed by dynamic activity to include stretching of the main muscle groups involved with the activity. The participants then performed the tests in the sequence they were familiarized with during their initial visit following a standardized warm-up (Figure 1).

### 2.5. Physical Performance Assessment

Physical performance was assessed using tests of muscular endurance (60% 1 RM to failure), strength (IMTP), and power (CMJ). For the muscular endurance tests, participants performed single leg press and shoulder press exercises using resistance machines (Technogym, Cesena, Italy). Participants performed as many repetitions as possible with correct form until failure, with provision of similar verbal encouragement across all trials. Muscular endurance tests of 60% 1 RM using resistance machines have been shown to be valid and reliable measures of endurance [30]. During the muscular endurance tests, participants estimated their rating of perceived exertion (RPE) using the Borg 6–20 scale [31] every three repetitions. The Borg 6–20 scale is a validated tool for assessing subjective perceptions of effort during exercise [32]. CMJ height (with arms placed on hips throughout the movement) was measured using an Opto Jump system (Microgate, Bolzano, Italy), previously shown as a valid and reliable tool for measuring jump height [33]. Mean jump height of three jumps was recorded to the nearest 0.1 cm. For the IMTP, peak force (N) was measured using force plates (Pasport, Pasco, Roseville, CA, USA) sampling at 1000 Hz. Participants performed the IMTP on an isometric testing apparatus at their preferred barbell positioning, ensuring bar height was consistent throughout all conditions with slight knee flexion evident. Force plates were zeroed prior to all bouts, and participants applied the minimum amount of pre-tension to allow for a stable force trace prior to the maximum pull [34]. Participants were instructed to pull the barbell with maximal effort for 5 s, maintaining the correct form throughout the test following a ‘3, 2, 1, pull’ verbal instruction. This was repeated for three separate measures. The IMTP has been shown to be a valid and reliable measure of maximal strength [35].

### 2.6. Statistical Analysis

Statistical analyses were performed using SPSS Statistics version 31 (IBM Corp., Chicago, IL, USA). Shapiro–Wilk tests assessed normality of physical performance measures. Normality results indicated that the data were normally distributed for all outcome measures across conditions, except for leg press repetitions with caffeine gum. As most of the data were normally distributed and considering the robustness of ANOVA to minor violations of normality, one-way repeated measures ANOVAs were performed for each physical performance outcome [36], with Greenhouse–Geisser corrections applied when sphericity was violated [37,38]. For the RPE data, two-way repeated measures ANOVAs were conducted to compare the effects of the conditions on RPE repetition for single leg and shoulder press. Effect sizes were calculated and interpreted based on Cohen’s guidelines [39]. A priori power analysis indicated a minimum of 12 participants to detect a 10% difference in muscular endurance with a power of 0.80 and an alpha of 0.05, based on previous investigations [40,41]. In addition to the group data, individual responses were evaluated using a ‘least significant difference (LSD)’ procedure, previously used within the literature [42]. The LSD was calculated using the pooled SD for each condition, with a beta value of 0.8, and alpha value of 0.05. The LSD reflects the minimum difference between 2 values (in this case, placebo vs. pouch or placebo vs. gum) that constitutes a significant difference. The rationale for the additional individual analysis is due to the variability in caffeine effects between individuals, which can be lost when reporting population-based analysis.

## 3. Results

**Figure 2:**
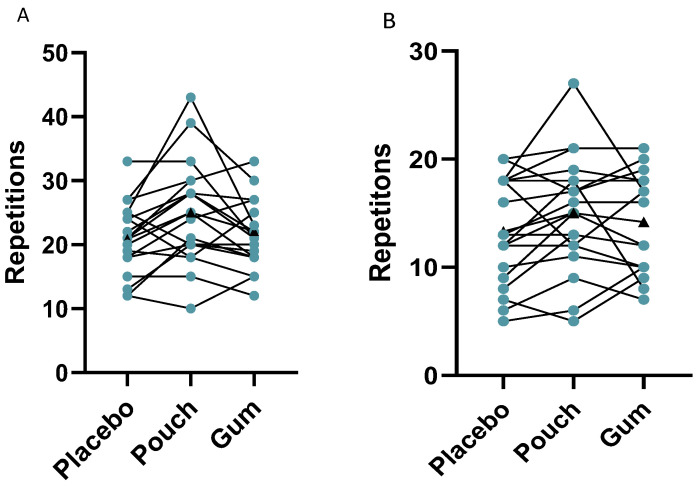

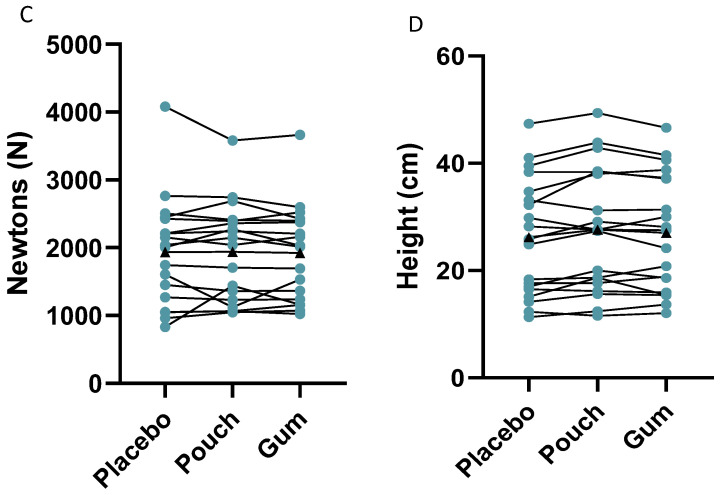
Individual data highlighted for the (**A**) leg press, (**B**) shoulder press, (**C**) IMTP, and (**D**) CMJ. Mean values for each condition are highlighted with black triangles.

Data for the IMTP test is reported for *n* = 18, as an equipment fault resulted in one condition not recording for a participant; therefore, this participant’s data was removed for all conditions. Further, an outlier was removed from the leg press data that reduced the number of participants in that analysis to *n* = 18; this exclusion was a result of visual inspection and did not affect the overall significance, with analysis revealing no significant main effects of condition on single leg press repetitions (*p* = 0.169). Percentage improvements for muscular endurance tests are shown below, with data including the outlier added in parenthesis. Similarly, the analysis revealed no differences for shoulder press repetitions (*p* = 0.100), IMTP average peak force (*p* = 0.945), or CMJ average height (*p* = 0.524). However, following the administration of the caffeine pouch and caffeine gum, improvements in muscular endurance for the single leg press were 19.0% (17.5%) and 5.6% (13.9%) respectively compared to the placebo gum, with improvements also observed for the shoulder press of 12.0% and 6.6% for the pouch and gum respectively. Figure 2 highlights the data from these measurements.

**Figure 3:**
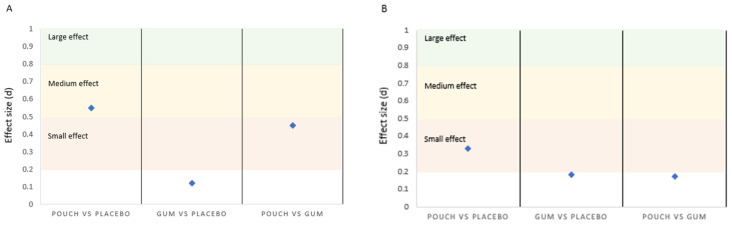
Effect sizes highlighted for the (**A**) leg press (*n* = 18) and (**B**) shoulder press (*n* = 19), suggesting potential meaningful results.

Effect sizes for the non-significant differences between conditions ranged from small to medium (0.01 to 0.55) based on benchmarks [36]. The effect sizes have the potential to be meaningful for the leg and shoulder press, with small and moderate effects compared to the placebo [38,43], suggesting potential practical significance despite the lack of statistical significance (Figure 3).

Table 1 highlights individualized results based on LSD analysis. For interpretation, a positive responder indicates that the participant benefitted from the treatment at or greater to the magnitude necessary to be considered significant (based on calculated LSD). A negative responder indicates significantly worse performance, and a non-responder indicates that the difference failed to breech the LSD value. For the single leg press to failure, the caffeine pouch demonstrated that 9 of the 18 participants were positive responders, 7 were non-responders, and 2 were negative responders, with similar results for the caffeine gum, with 7 of the 18 participants responding positively, 8 were non-responders, and 3 were negative responders.

For the shoulder press to failure, the results indicated that 8 of the 19 participants were positive responders, 9 were non-responders, and 2 were negative responders. Limited effects were observed for the caffeine gum, with 4 positive responders, 14 non-responders and 1 negative responder. Only one positive responder was observed for the IMTP for the caffeine pouch, with the rest of the participants being non-responders with that activity, and similar results were obtained for the CMJ, with all participants being non-responders.

**Figure 4:**
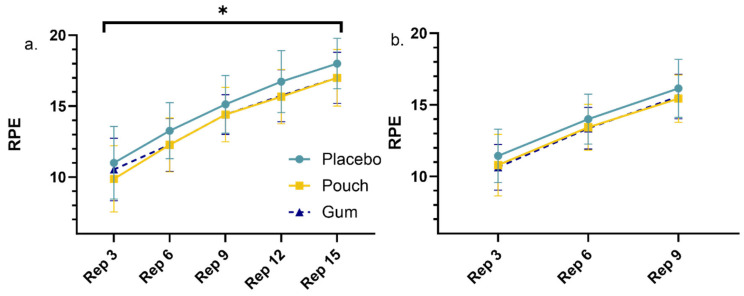
RPE measured follow every 3rd repetition to failure during the leg press (**a**) and shoulder press (**b**). * Indicates significant condition effect, with post hoc analysis identifying significance between the placebo and caffeine pouch (*p* = 0.032).

Further to the performance measures, RPE values were reported every third repetition for comparison between conditions (Figure 4). For the leg press, the number of repetitions that most participants reached before failure was 15 reps (*n* = 15); therefore, every third repetition up to 15 was included. The analysis highlights a significant main effect for RPE and condition (*p* = 0.022), with least significant difference post hoc analysis identifying significance for the placebo vs. pouch conditions (*p* = 0.032) and a statistical trend for the placebo vs. gum conditions (*p* = 0.060). Both showed higher RPE with the placebo. No significance was observed for the pouch vs. gum (*p* = 0.582). Significance was also observed for repetition (*p* < 0.001), with RPE increasing with repetition in a linear fashion. No interaction effect was identified (*p* = 0.402). For the shoulder press, no significant condition or interaction effect was observed (*p* = 0.094; *p* = 0.209), although, as expected, a repetition effect was seen (*p* < 0.001).

## 4. Discussion

The primary aim of this investigation was to explore the effects of a low dose of caffeine (80 mg) administered in novel formats on physical performance in recreationally active adults. In comparison to the placebo condition, it was hypothesized that the low dose of caffeine would significantly enhance physical performance, with the ergogenic effects observed regardless of the delivery method (gum or pouch). Based on aggregate data analyses, the findings show no statistically significant differences in physical performance among conditions. However, of importance is the fact that individual responses highlight the variability in caffeine and the overall positive effect that is possible. Of further interest is the fact that RPE was significantly reduced with the administration of the caffeine pouch following post hoc least significant difference analysis during the leg press exercise.

Performance outcomes show no significant differences among the placebo, pouch, and gum conditions, although a statistical trend was observed for the pouch over the placebo during the single leg press. These findings contradict previous research that has demonstrated ergogenic effects of caffeine on physical attributes [39,44,45], although it is important to recognize the dosage between investigations, with recommendations currently set at 3–6 mg/kg BM [3], and the majority of research is guided by this. Indeed, previous research with caffeinated gum observed improvements in lower limb strength and power, in addition to specific peak power in rowing, with an absolute dose of 300 mg, which equated to a relative dose range between 2.7–4.5 mg/kg BM [46]. The current investigation provided a mean relative intake of 1.1 mg/kg BM (80 mg absolute), which may not have elicited an ergogenic/significant response. There was also a large variation in body mass (72.8 ± 16.9 kg) between participants, and the use of the absolute dose from the caffeine pouch and gum to maintain an ecological approach may have compounded this further. Administering absolute caffeine doses with caffeinated gum is not uncommon, and observations have found significant enhancements for strength, although the range of the absolute dose within a contemporary review [24] was generally between 200–300 mg to elicit an ergogenic response, which is greater than the current investigation. However, there is a growing body of evidence to suggest that lower doses of caffeine can have positive benefits for sporting endeavors [47,48,49]. A meta-analytic approach conducted by Grgic [18] exploring the minimum ergogenic dose of caffeine on muscular strength, endurance and velocity concluded that the minimal ergogenic doses of caffeine are lower than previously suggested (0.9–2.0 mg/kg BM). In addition, the meta-analysis highlighted that the magnitude of these effects was similar to that previously reported with higher caffeine doses. Notwithstanding the lack of significance, the current investigation did find potentially meaningful effect sizes [43,50] in favor of the administration of caffeine for muscular endurance. These findings, as an enhancement to the non-caffeine condition, show medium effect sizes of Cohen’s d = 0.55 for the caffeine pouch for the leg press and a small effect of d = 0.33 in favor of the caffeine pouch vs. the placebo for the bench press (Figure 3). These are in agreement with a review by Polito and colleagues [51], who observed an overall effect size of d = 0.38, with a range between d = 0.29–0.48, although the majority of the investigations included in the review administered a caffeine dose between 4–6 mg/kg BM. The meta-analysis [17] with lower caffeine doses observed a small effect size of d = 0.21, with a range of d = 0.07–0.35. This review article would seem to suggest that larger caffeine doses elicit a greater effect, with the current investigation contradicting that view, although there is further support from a comprehensive investigation comparing a low (2 mg/kg BM), moderate (4 mg/kg BM), and high (6 mg/kg BM) caffeine dose, with findings suggesting no dose response effect and effect sizes (d = 0.46–0.68) [47] for lower-body muscle endurance, which is comparable to the leg press in the current investigation.

These small and medium effect sizes highlighting the increased repetitions to failure may be facilitated by a reduction in perceptual ratings at the same given intensity/load, which are mediated by the administration of caffeine. Indeed, RPE was lower following caffeine consumption, which resulted in 19.0% and 12.0% increases for the caffeine pouch during the single leg and shoulder press to failure respectively and increases of 5.6% and 6.6% for the caffeine gum, signifying a benefit of caffeine consumption during this activity. The reductions in RPE observed in the current investigation were statistically significant during the leg press exercise, with post hoc analysis identifying a significantly dampened RPE response with the caffeine pouch compared to the placebo gum and statistical trends for the caffeine gum lowering RPE compared to the placebo gum. Although many mechanisms have been proposed and include increased myofibrillar calcium availability, increased motor unit recruitment, and improved excitation–contraction coupling [3,17,43], due to the lower caffeine dose administered in the current investigation, physiological changes may not have been present. Nevertheless, ergogenic effects have been observed, with low caffeine doses showing strong support purported to the primary mechanisms of action that caffeine stimulates the central nervous system (CNS) and its antagonism of adenosine receptors [3]. The systematic review by Yang and colleagues [24] agrees with this notion and suggests that strength improvements observed with low caffeine doses are more likely due to this sympathetic nerve activity, with adenosine inhibition augmenting neuronal excitability and enhancing catecholamine levels that increase heart rate, blood pressure and muscle tone. Furthermore, awareness has grown on the action of caffeine during exercise within the central and peripheral nervous systems providing an enhanced capacity to tolerate pain and fatigue [52]. Within the current investigation, a caffeine-mediated decrease in RPE may have allowed for more repetitions, which is also supported within the literature [9,53,54]. However, a dampened RPE response is not universally observed [55,56,57]. Suggestions on this disparity may be based on the timing of the measurements. Indeed, investigations that have not seen a reduction in RPE following caffeine administration generally only report at exercise termination, with perception throughout the exercise missed. Investigations that have reported RPE throughout exercise [45,58] have observed similar results to the current investigation. These differences and trends identified in RPE with caffeine may hold practical implications for athletes and coaches. Higher perceived exertion during training and competition could potentially influence pacing strategies, decision-making, and overall performance [59]. Therefore, observing individual responses to caffeine supplementation by means of monitoring and reporting RPE could provide valuable insights for optimizing performance outcomes in training and competition [60].

As there are interindividual responses to caffeine consumption [3], relying exclusively on group analysis may obstruct uncertain conclusions. The current investigation added to the aggregate analyses a more individualized approach using the “least significant difference” to identify which participants responded more notably to caffeine during the physical tests. This approach, used previously with a micronutrient supplementation [42], provides a more thorough analysis by clarifying how many participants respond to a degree that can justifiably be viewed as a significant/meaningful result. Within this analysis, it highlights that 50% of participants increased their repetitions with the caffeine pouch over the placebo and therefore responded positively to the caffeine pouch. Similar trends were identified with caffeine gum, with 39% responding positively. Following the shoulder press tests, the caffeine pouch observed a 42% positive increase, with 21% positive responders with the caffeine gum. The reduced positive effect with the shoulder press compared to the leg press may be expected, as previous research [52] suggests a lower baseline muscle activation level provided more scope to improve, whereas the smaller upper-body muscle groups have a much higher activation baseline. These results highlight that although significant results may not be achieved at a group level, many participants gain ergogenic benefits that may be more advantageous within an athletic setting for performance purposes. While analysis of group means is needed, the interindividual variability that inevitably exists with caffeine requires assessment of participants as individuals to most effectively assess treatment efficacy. From an application standpoint, if caffeine benefits an individual, the lack of overall statistical significance for group means does not negate said benefit. Further research is warranted to identify the reasons for variable responses.

The current investigation is not without its limitations. Genetic variations in caffeine metabolism, such as polymorphisms in the CYP1A2 gene, have been shown to affect individual responses to caffeine [3,19,61], with individual differences in caffeine sensitivity and tolerance influencing the response to acute caffeine supplementation [62,63]. Therefore, future studies could potentially consider genotyping participants to account for these genetic differences in caffeine sensitivity and metabolism. Similarly, it may be beneficial to record caffeine habituation. The rationale for its exclusion in the current investigation was due to the contemporary literature suggesting that performance is not influenced by habitual caffeine consumption [64,65], although differences in habituation may influence participants’ ability to distinguish between the placebo and caffeine conditions and the perceived effects. Moreover, the investigation did not measure plasma caffeine concentrations or assess the time course of potential effects. Incorporating these measures in future research could provide valuable information about the pharmacokinetics of low-dose caffeine and the optimal timing of administration for performance benefits, especially for the caffeine pouch, as this analysis has not been investigated. Finally, the lack of a placebo pouch needs to be highlighted, and this raises the potential placebo effect associated with caffeine supplementation. As no placebo pouch was included and participants were aware of the caffeine content, there could be an additional source of bias, as participants’ expectations and beliefs about the effects of caffeine can significantly impact their performance [66]. Future studies should endeavor to include placebo condition for all products and systematically measure their blinding success. A double-dissociation design [67,68] could be employed to control for placebo effects and isolate the true effects of low-dose caffeine.

## 5. Conclusions

In conclusion, despite the lack of significant improvement with physical parameters, the comparison of chewing gum and oral pouches in this investigation provides valuable insights into the practicality and potential use of these novel caffeine administration methods. Small and medium effect sizes are identified, with individual analysis supporting improvements with a high percentage of participants. Further, the dampened RPE response with the caffeine pouch is of interest. Caffeine chewing gum and oral pouches may offer convenient, discreet, and portable options for low-dose caffeine consumption. This could be appealing to athletes, students, or professionals looking to improve their physical performance throughout the day. Conversely, individual preferences and tolerance may play a role in the choice of delivery method. Some individuals may find chewing gum or oral pouches uncomfortable or unpleasant, while others may prefer these methods due to the avoidance of the GI tract. Future research could explore preferences and experiences with different low-dose caffeine delivery methods to inform practical recommendations more accurately.

## Figures and Tables

**Table 1:** Individual responses utilizing an LSD approach for strength endurance tests.

Participant	Leg Press (Response Based on LSD)		Shoulder Press (Response Based on LSD)	
	Placebo vs. Pouch	Placebo vs. Gum	Placebo vs. Pouch	Placebo vs. Gum
1	−	−	NC	NC
2	−	−	+	NC
3	+	NC	−	NC
4	NC	NC	NC	NC
5	+	+	+	NC
6	NC	NC	+	NC
7	+	+	−	NC
8	NC	+	NC	NC
9	NC	NC	NC	NC
10	NC	+	NC	NC
11	O	O	NC	+
12	+	NC	+	NC
13	+	+	+	+
14	+	NC	+	+
15	+	+	+	+
16	NC	NC	NC	NC
17	NC	−	NC	NC
18	+	NC	+	−
19	+	+	NC	NC

LSD = least significant difference; NC = no change/non-responder; + = positive responder; − = negative responder; O = outlier.

## Data Availability

The original data presented in the study are openly available in DOI: https://doi.org/10.15126/surreydata.901517.

## Abbreviations

The following abbreviations are used in this manuscript:

LP	Leg Press
SP	Shoulder Press
RPE	Rating of Perceived Exertion
CNS	Central Nervous System
RM	Repetition Maximum
IMTP	Isometric Mid-Thigh Pull
CMJ	Counter-Movement Jump
LSD	Least Significant Difference
